# Application of a Hyaluronic Acid Gel after Intrauterine Surgery May Improve Spontaneous Fertility: A Randomized Controlled Trial in New Zealand White Rabbits

**DOI:** 10.1371/journal.pone.0125610

**Published:** 2015-05-11

**Authors:** Stephanie Huberlant, Herve Fernandez, Pierre Vieille, Mohamed Khrouf, Daniela Ulrich, Renaud deTayrac, Vincent Letouzey

**Affiliations:** 1 Department of Obstetrics and Gynaecology, Nimes University Hospital, Nîmes, France; 2 Department of Obstetrics and Gynaecology, Hôpital de Bicêtre- Assistance Publique- Hopîtaux de Paris, Le Kremlin-Bicêtre, Paris, France; Queen's University, CANADA

## Abstract

**Objective:**

Intrauterine adhesions (IUAs) are the most common complication after hysteroscopy in patients of reproductive age. Intra-abdominal anti-adhesion gel reduces the incidence of adhesions, but effects on fertility after uterine surgery are not known. The objective of our work was to evaluate the effect of intrauterine anti-adhesion gel on spontaneous fertility after repeated intrauterine surgery with induced experimental synechiae in the rabbit model.

**Materials and Methods:**

Twenty New Zealand White rabbits underwent a double uterine curettage 10 days apart and were randomized into two groups. Each rabbit served as its own control: one uterine tube was the treatment group (A), the second uterine tube was the control group (B) to avoid bias through other causes of infertility. Group A received a post curettage intrauterine instillation of anti-adhesion gel whereas group B, the control group, underwent curettage without instillation of the gel. After a recovery period, the rabbits were mated. An abdominal ultrasound performed 21 days after mating allowed us to diagnose pregnancy and quantify the number of viable fetuses.

**Results:**

There was a significant difference in total fetuses in favor of group A, with an average of 3.7 (range, 0–9) total fetuses per tube against 2.1 (0–7) in group B (p = .04). The number of viable fetuses shows a trend in favor of group A, with an average of 3.4 (0–7) viable fetuses per tube against 1.9 (0–6) viable fetuses per tube in group B (p = .05).

**Conclusion:**

The use of immediate postoperative anti-adhesion gel improved fertility in an animal model after intrauterine surgery likely to cause uterine synechiae. This experimental model will permit comparison of different anti-adhesion solutions, including assessment of their tolerance and potential mucosal toxicity on embryonic development.

## Introduction

Uterine synechiae consist of the partial or total (Asherman's syndrome) apposition of the walls of the uterus and are the most common complications after operative hysteroscopy in patients of reproductive age [1,2]. The incidence of uterine synechiae depends on the type of surgery. Taskin *et al*. [3] describe postoperative adhesions rates from 4% for polyp resection, to 6.7% for uterine septum resection and up to 45% after resection of multiple myomas. In one study of infertile women, the incidence of intrauterine adhesions (IUAs) was 10% [4] and the prevalence of post miscarriage adhesions has ranged from 8–37% in different studies [5]. Adhesions appear on average between 4 to 6 days after surgery.

Degrees of adhesions can be classified into three groups according to the ostial visibility [6]. Asherman's syndrome is the most severe manifestation; it consists of complete obstruction of the uterine cavity and may cause secondary amenorrhea [3,7]. The symptoms of Asherman’s syndrome and IUAs are variable, the most common being dysmenorrhea, secondary amenorrhea, infertility and spontaneous abortion. There can be obstetric complications with higher rates of placenta praevia or accreta in patients with a history of uterine resection followed by adhesions [8].

Synechiae are diagnosed on clinical call points or they may be asymptomatic and discovered incidentally, such as when infertility is being investigated. Hysterography and hysterosonography examinations are quite useful and particularly sensitive to highlight intrauterine anomalies; however, they are not sufficiently specific for the diagnosis of uterine synechiae [9]. Hysteroscopy is currently the gold standard for diagnosis of adhesions and is also used for therapeutic purposes [8]. In fact, it is recommended to perform a hysteroscopy to observe the appearance of the uterine cavity after surgery and remove any adhesions [6,7,10]. To improve the prognosis of both the primary and secondary infertility that is linked to IUAs, it is essential to understand why adhesions develop [7,11].

The use of intra-abdominal anti-adhesion gel has been evaluated in women and this appears to improve the prevention of those postoperative adnexal and digestive tract adhesions which entail a risk of infertility [12,13]. Several authors have studied the mechanical impact of anti-adhesion gel on abdominal adhesions, showing a decreased risk of postoperative adhesions [11–14]. Guida *et al*. have demonstrated the efficacy of a hyaluronic acid gel for the primary prevention of adhesions in women after hysteroscopic resection of intrauterine synechiae [15], but effects of this gel on fertility were not reported. Human studies to date have not shown a significant increase in the rate of live births, regardless of the type of anti-adhesion agent used [16–18]. Hence we propose that further research into substances that could prevent infertility due to uterine synechiae is warranted [16,17].

Animal models such as the rabbit are useful in the study of fertility disorders, including those involving uterine abnormalities [18,19]. Indeed, the female rabbit has a bicornuate uterus where 2–7 fetuses can develop on each side. This anatomical specificity allows the use of each animal as its own control. The aim of our study was to evaluate, in the rabbit model, the impact on fertility of anti-adhesion gel after curettage. The primary endpoint was the number of viable fetuses 21 days after mating.

## Materials and Methods

### Animal Welfare

This is a randomized experimental study conducted on 20 female New Zealand White rabbits, weighing between 2500 and 3500g and being 15 weeks old at the beginning of the study. The animal surgery was done at the University of Montpellier-Nimes, in agreement with the regional ethics committee (Comité d’éthique pour l’expérimentation animale Languedoc-Roussillon/Animal Care and Use Committee Languedoc-Roussillon) for animal experimentation (Reference No. CEEA-LR-11019). This research laboratory has all the necessary approvals for this type of animal research. All experiments were conducted in accordance with good practice for the use of animals according to the Animal Welfare Act. Our animal housing and surgical procedures were based on the rat uterine horn model of *Kaya et al* [20]. The 20 rabbits were housed in single cages with a 12 hr cycle of light and dark, and ad libitum access to a standard rabbit pellet diet and water. The animals were anaesthetised by an intramuscular injection of 16 mg xylazine to minimize suffering, followed 5 min later by an intravenous injection of 20 mg ketamine hydrochloride. At the end of the operation, a 12-mg/h fentanyl patch (Mylan Pharmaceutical) was placed on the skin for 72 hours for postoperative analgesia. At the end of the experiments the rabbits were humanely euthanized using barbiturate.

### Description of the study

**Fig 1:**
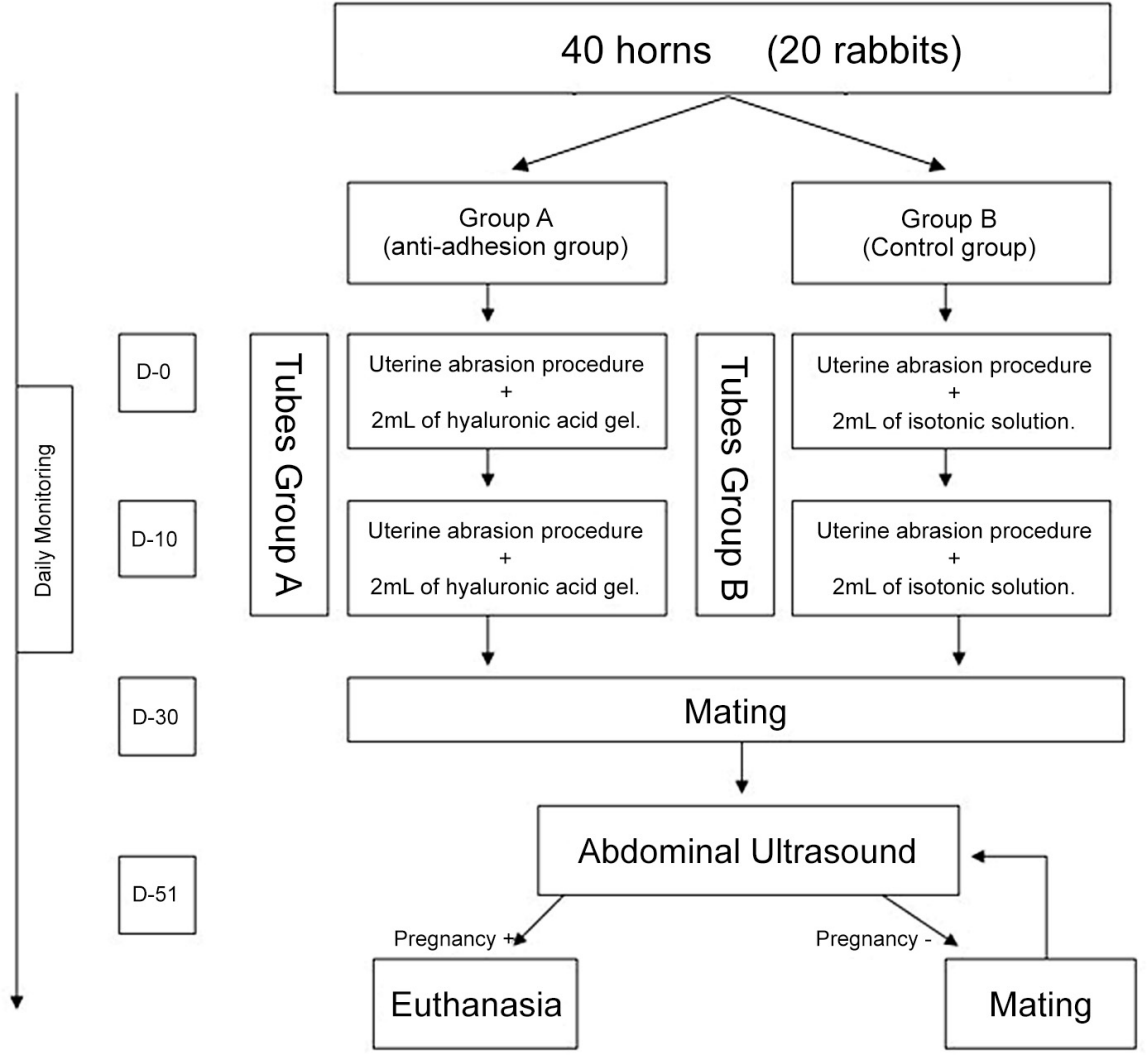
Flow chart of the experimental protocol. 20 rabbits/ 40 uterine tubes were divided into a test (Group A) and control group (Group B).

Rabbits have a bicornuate uterus and embryonic development takes place in the uterine tube. Therefore we analyzed the results according to the tubes and not in individual animals, since each animal was its own control: The anti-adhesion gel was instilled only in one side of the uterine tube while the saline was instilled into the otheruterine tube. Female rabbits were mated with three male animals to limit bias due to male infertility. After 72 hours of acclimatization, the 20 rabbits underwent a double bilateral curettage; both uterine tubes were curetted at the same time in each animal. For Group A uterine tubes, 2 mL of anti-adhesion solution was instilled immediately after curettage. The other uterine tube in that same animal was the control (Group B) uterine tube, into which 2 mL of isotonic saline was instilled immediately after curettage. The uterine tubes of each animal were randomly allocated for instillation of the anti-adhesion solution or saline. The experimental timeline is illustrated in Fig 1.

### Description of the gel

Different methods for prevention of tubal adhesions have been developed for intra-abdominal use [6]. There are few comparative randomized studies of anti-adhesion agents in the abdominal model [21], and none in animal models of IUAs. The difficulty of intrauterine instillation has limited assessment of the hyaluronic acid agents recently developed for the prevention of intra-abdominal adhesions. We chose hyaluronic acid gel (Hyalobarrier) due to its recent approval for clinical use in our university hospital. Moreover, it is a quite new product and mostly used to prevent intra-abdominal adhesions and complications.

### Surgical procedure

The rabbit model of intrauterine synechiae has been developed in previous work [19]. The synechiae develop secondarily to an abrasive curettage of all sides of the uterus with a 3 mm curette.

On Day 0, the females were anaesthetised by an intramuscular injection of 16 mg xylazine (Rompún, Bayer AG, Leverkusen, Germany) to minimize suffering, followed 5 min later by an intravenous injection of 20 mg ketamine hydrochloride (Imalgène, Merial SA, Lyon, France) to maintain anaesthesia during the laparotomy. The surgical procedures were performed under sterile conditions. All operations were performed by the same researcher who was blinded to the treatment group. The uterine tubes were randomly assigned and not sequentially operated on, in order to minimize bias. The rabbits were shaved in the abdominal region and a sterile drape was placed at the operative site after disinfection with polyviode iodine. A 3-cm vertical midline incision was performed and both uterine tubes were exposed. The peritoneal opening allowed access to the abdominal cavity to ensure the absence of organic disease in the reproductive tract (uterus, oviducts, and ovaries).

**Fig 2:**
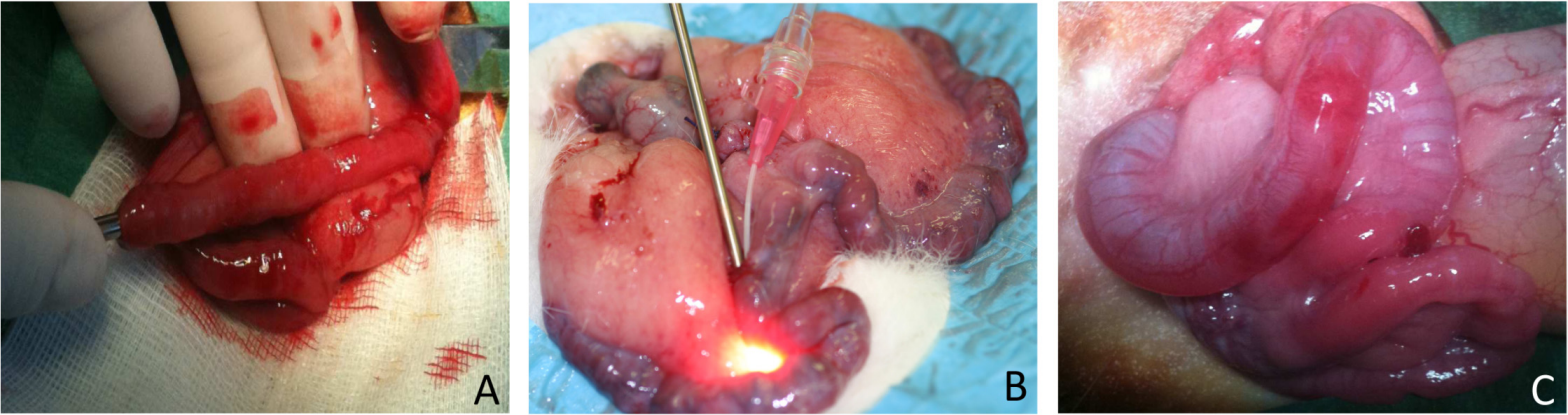
Operative procedure of the rabbits. A. Tubal curettage with a 3 mm curette; B. Instillation of 2 mL anti-adhesion solution; C. Rabbit bicornuate uterus: the left uterine tube is dilated by the instillation of 2mL anti-adhesion solution.

The uterus was externalized for the procedure and curettage was performed after tubal incision with scissors on ¼ of the tubal circumference, approximately 1 cm from the uterine tube at the distal third of the tube. A 3 mm curette inserted through this incision allowed curettage by intratubal surface abrasion for 1 minute (Fig 2A) as described previously [22].

For Group A tubes, 2 mL of anti-adhesion gel was instilled via a tubal catheter (Fig 2B). The administration of anti-adhesion gel was performed just after tubal curettage. Control tubes in Group B received the same volume of isotonic saline, also via a tubal catheter. The surgical procedure and the instillation of anti-adhesion solution led to some bleeding, and adequate hemostasis was achieved by simple compression of the incision point.

The abdominal incision was closed in two layers using a simple interrupted 4–0 polyglactin 910 suture for the peritoneum–fascia and for the skin. The rabbits were placed in individual cages and were monitored daily. Ten days after this procedure, a second bilateral tubal abrasive curettage, in order to ensure adhesion was performed in these 20 rabbits according to the procedure described above. The anti-adhesion gel was instilled in one uterine tube and the isotonic saline in the other uterine tube. Intratubal adhesions were checked during a third laparotomy using hysteroscopic visualization of the inside of the tube. Based on our previous results synechiae are present in all animals after 2 curettages. The timeline for this set of experiments is illustrated as Fig 1. 30 days after the first surgical procedure (Day 30), the females were mated with a reproductive age male, for evaluation of the effect of the surgery and treatment on their ability to conceive and gestate. In female rabbits, coitus causes release of the eggs. The gestation period is usually 31–32 days, but can vary between 29 and 35 days [23]. To limit the potential for bias due to male infertility, three New Zealand White male rabbits were selected and randomly used for breeding.

### Diagnosis of pregnancy

The diagnosis of pregnancy was made by abdominal ultrasound 21 days after mating. The non-pregnant rabbits were mated again and a new ultrasound was performed in the next cycle.

If pregnancy was diagnosed, animal euthanasia was performed for clinical analysis to confirm the number of pregnancies visually in the uterine tubes. Non- viable fetuses can be distinguished due to their smaller size at time of abdominal exploration. Euthanasia of animals was induced by pentobarbital lethal injection, as instructed by our local ethics committee.

### Statistical analysis

The number of animals included was determined after reviewing the literature [17,19]. The number of animals required was determined according to a calculation with a power of 90%, an alpha risk of 5% and an odds ratio (OR) of 10 (3.7% → 37) following unilateral chi^2^ or Fisher’s tests [17,20]. N = 20 animals (i.e. 40 tubes) were considered necessary covering a loss of up to 2 animals.

The collected data were analyzed and compared. The number of viable fetuses for each tube was noted in each group and analyzed using the Student t-test. This was chosen in order to obtain, in a homogeneous population of normal distribution, a quantitative answer of independent factors (number of fetuses). Group comparisons were made using the Student's t-test or, when the data were not normally distributed, the Mann-Whitney U test. Tests of significance were two-sided with a 0.05 alpha level. All data analyses were performed with SAS software version 9.2 (SAS).

## Results

Twenty rabbits underwent two rounds of curettage, with random allocation of one uterine tube for intratubal instillation of anti-adhesion gel, and instillation of saline into the other uterine tube of that same animal. The repeated anesthesia for the confirmation of intratubal adhesions beared severe complications (3 animals died) thus the operation was only performed in those 3 animals. Intratubal adhesions were present in all animals with at least 50% of the intratubal lumen being covered by adhesions. The overall observation period was 51 days. During this period, three deaths occurred. These deaths occurred during induction of anaesthesia. During the post-surgical monitoring period, we tracked body weights and observed an average weight gain of 40% above the initial weight.

**Fig 3:**
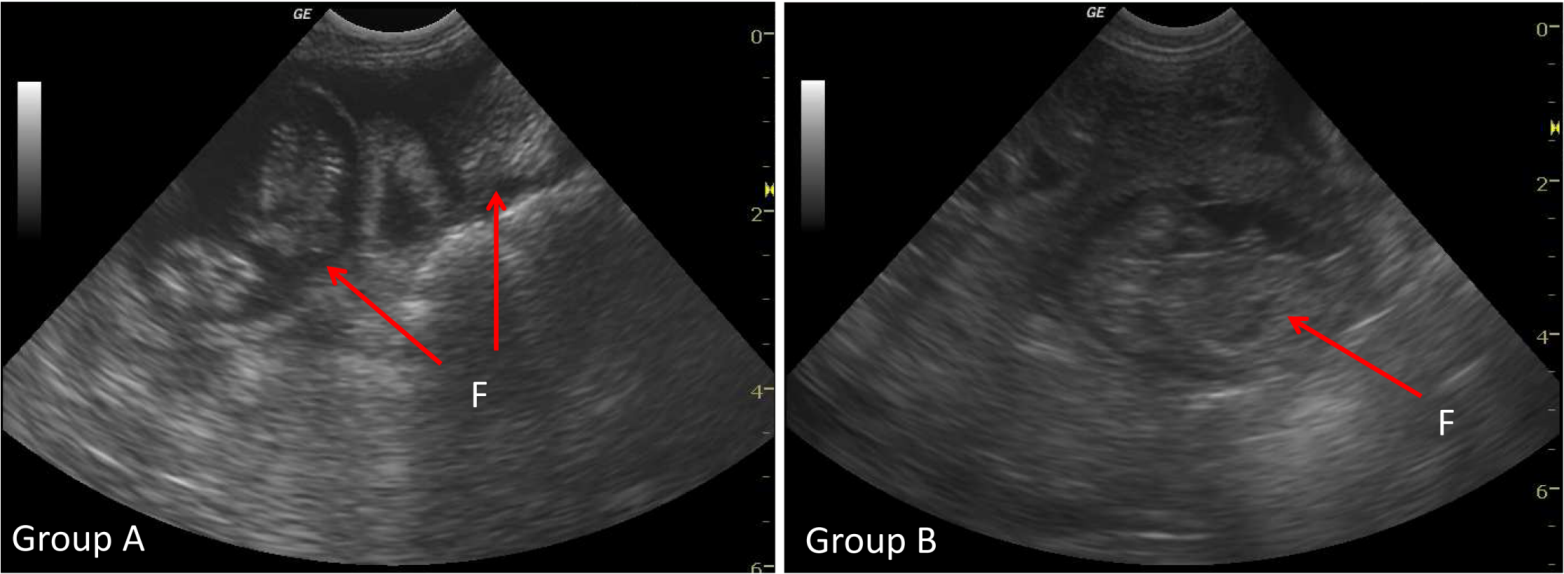
Ultrasound pregnancy diagnosis. At Day 21 post mating, by counting the number of foetuses (F). Test (Group A) and control group (Group B).

When the test substance was instilled, we observed an expansion of the tube receiving the anti-adhesion solution (Fig 2C). Fetuses were identified by ultrasound, 21 days after mating and confirmed secondarily by animal euthanasia, following the experimental practices guide, and counting the number of viable fetuses in each tube (Fig 3). 88.2% of females were pregnant after the first mating, and all females were pregnant after the second mating.

Results for pregnancy outcomes are shown in Table 1. Calculations are based on 17 uterine tubes per group, due to the death of three animals. There is a significant difference for the number of fetuses in favor of Group A, with an average of 3.7 (range, 0–9) total fetuses per tube against 2.1 (0–7) in Group B (*P* = .04). The rate of viable fetuses was higher in Group A, which received postoperative instillation of anti-adhesion gel with an average of 3.4 (0–7) viable fetuses per tube in Group A, against 1.9 (0–6) viable fetuses per tube in Group B (*P* = .05).

**Table 1:** Pregnancy data.

	Group A (Anti-adhesion Group)	Group B (Control group)	P value
Uterine tube model (number)	17	17	
Total initial fetuses	63	36	p< 0.05
Average total fetuses (range)	3.7 [0–9]	2.1 [0–7]	p< 0.05
Number of pregnancy losses	6	3	p = 0.09
Average viable fetuses at term	3.4	1.9	p = 0.05

Comparison of number of fetuses between the test (Group A) and the control (Group B) uterine tubes.

We performed hysteroscopy on three animals, before mating, and observed IUAs in all three. It was not practical to perform hysteroscopy on all animals due to the death’s risks of a third round of anaesthesia in the space of one month.

## Discussion

Devices to prevent postoperative intra-abdominal adhesions have previously shown their effectiveness in terms of reduced postoperative pain and complications [7,21]. Some teams have recently studied the impact of intrauterine anti-adhesion solution in animal models [9,15,17]. This led us to evaluate the anti-adhesion gel using a rabbit model of double tubal curettage that is associated with post-surgical intrauterine adhesions. A randomized study on the intrauterine use of carboxymethylcellulose sodium associated with polyethylene oxide (Intercoat) showed a significant reduction of postoperative adhesions [18], but the impact on spontaneous fertility was not studied. Hence we studied if hyaluronic acid gel was effective in preventing IUAs in rabbits, and we chose the number of viable fetuses to be the main endpoint of our study.

We report that anti-adhesion gel improves spontaneous fertility in rabbits undergoing curettage, since the number of viable fetuses was almost doubled in comparison with our control group. This is a significant update on the work of De Iaco *et al*. (1998) who described a 50% decrease in postoperative adhesions in a rabbit model of intrauterine adhesions treated with a hyaluronic acid solution, but the impact on spontaneous fertility was not studied [24].

The validation of a barrier anti-adhesion agent requires evaluation in a pre-clinical animal model. The rabbit tubal curettage model allows use of each animal as its own control, therefore overcoming potential physiological bias between animals. The choice of the rabbit model was based on the literature, as rabbit reproduction is well understood and easily managed. The animal model was already described [24] and rabbits are much less costly to study than larger animals such as sheep. In a previous study we showed that sufficient IUA are present when two curettages 10 days apart are performed while this is not the case for a single procedure [22]. Although the rabbit fetus develops in the uterine tubes, the presence of multiple fetuses per litter and the short duration of gestation were favorable to the establishment of the protocol. As a future refinement of the model additional local anesthesia and earlier application of the Fentanyl patch (twenty four hours before surgery) should be considered. Alternatively, Tramadol could be given prior to the incision to cover until the Fentanyl begins to provide sufficient analgesia.

An interesting and clinically relevant advantage of this procedure was the ability to check tubal patency intraoperatively by observation of tubal dilatation during anti-adhesion gel instillation. The 51 day period of monitoring and procedure was necessary to obtain a satisfactory recovery between each step so that the mating was not limited by the postoperative healing period [23].

No study until now has determined the impact of hyaluronic acid solutions on embryonic development. The different results in terms of total and viable fetuses could be due to the intratubal additive disturbing the uterine lining or embryonic development (during the implantation phase). The rates of pregnancy loss were similar between both groups and within the typical range for rabbits so a specific effect of the gel on pregnancy loss is unlikely [25]. A comparison between a control group which received saline infusion and a group that receives an instillation of gel without tubal curettage could address this question in future studies. Similarly, animal studies which precisely analyze the consequences of anti-adhesive agents on embryonic development, reinforced by histological analysis of tubal samples, could reveal the mechanism of involvement of these agents on changes in the tubal mucosa and their impact on implantation. Furthermore, in the context of secondary treatment of adhesions, it would be interesting to observe the effects of using hyaluronic acid *after* surgical removal of adhesions. This would help us to understand if the mechanism of these agents lies in preventing the formation of adhesions.

The effectiveness of different anti-adhesion agents was evaluated by Binda *et al*. using an intra-abdominal model in mice [26]. It would be interesting to adapt such comparative analysis to an intrauterine model, with spontaneous fertility as the primary endpoint. Comparing the effectiveness of different agents could help to understand the complex pathophysiology of potential toxicity before clinical study in women of reproductive age. Indeed, it would be worthwhile to establish the specific mechanism of adhesion development after intrauterine surgery, and how these adhesions impair fertility. The rabbit model that we have used and refined in this study is ideal for the in depth study of the development, treatment and prevention of infertility after uterine injury.

## Conclusion

The use of immediate postoperative anti-adhesion intrauterine solution improved spontaneous fertility in rabbits that underwent curettage. This experimental approach could be used to compare different anti-adhesion solutions, assess their tolerance and their effect on embryonic development, implementation, and development and quality of the endometrium before any study or use in women of reproductive age. Our results may contribute to improving spontaneous fertility of women after intrauterine surgery in the future.
